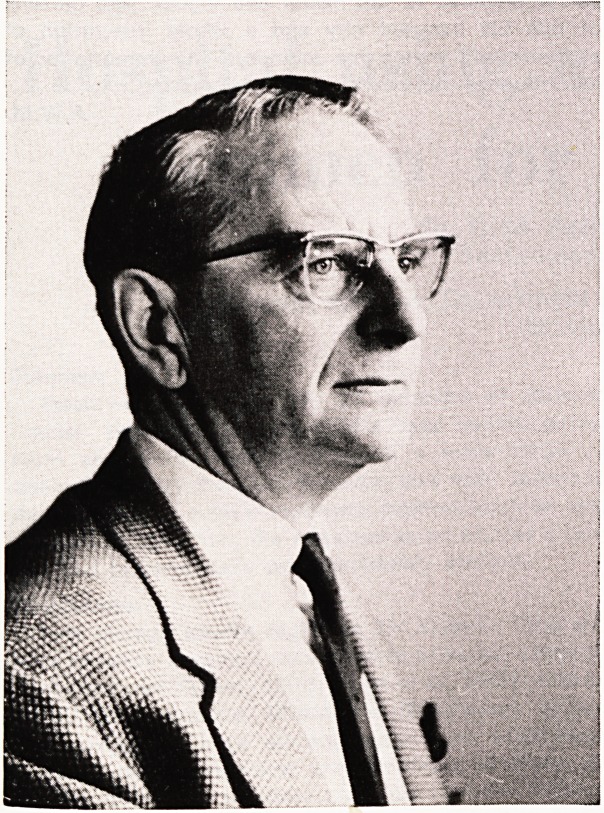# R. C. Wofinden

**Published:** 1976

**Authors:** 


### OBITUARY

**R. C. WOFINDEN, M.D., F.R.C.P., F.F.C.M., D.P.H., D.P.A.**

Robert Cavill Wofinden was born in the West Riding village of Greasbro on 25th January 1914. From Rotherham Grammar School he won a county major scholarship to St. Mary's Hospital, where his prizewinning notoriety was the despair of his peers. He was also a keen sportsman: a cricketer and golfer of parts, and soccer captain of St. Mary's. He graduated with honours as Alexander Fleming Prizewinner in 1937, and in 1938 he married Eileen Frances Rachel Sinnamon. In 1939 he proceeded to his Doctorate and took his Diploma in Public Health with honours at the London School of Hygiene and Tropical Medicine. He added the Diploma in Public Administration to his armoury in 1945, and was successfully elected Member and Fellow of the Royal College of Physicians in 1967 and 1972, in which year he also became a Founder Fellow of the Faculty of Community Medicine, with whose early origin he was closely concerned.

In 1939 he took up his first public health appointment in Rotherham, and began his classic field work on problem families. In 1946 he won the Joseph Rogers Prize of the Society of Apothecaries for an essay on Health Services in England, and moved to Bradford as Deputy Medical Officer of Health. A year later he became deputy to Robert Hughes Parry in Bristol, where he was to devote the remaining 27 years of his professional life, succeeding Dr. Parry as Medical Officer of Health of the City and County and Professor of Public Health in the University of Bristol in 1956. Here he continued his seminal studies of problem families, which were published by the Eugenics Society and earned him the rare distinction of its Honorary Fellowship. He consolidated and enhanced Bristol's international reputation as a model for the administration of health services and as a proud pioneer of imaginative innovations, and made the name of the city synonymous with health centres a decade before they became the fashion. Simultaneously he patiently developed the University department as the academic counterpart of his City department, and attracted students from all quarters of the globe.

Nationally, Robert Wofinden applied his great talents and experience to a notable range of work, including the Cohen Committee on Health Education, the Porritt Committee which foreshadowed reorganisation of the National Health Service, the training of public health nurses, and housing policy.

Following a World Health Organisation Fellowship to study health and social services in Scandinavia in 1954 he quickly gained international stature as a W.H.O. Consultant in a wide area of fields.

In later years he crystallised his expert views on many appropriate occasions. In 1967 "Health Centres and the General Medical Practitioner" was the theme of his Malcolm Morris Memorial Lecture, and "International Comparisons of Mortality" the topic of his Long Fox Memorial Lecture. In 1969 he discussed "Strategy and Tactics for the Public Health Service" in his presidential address to the Society of Medical Officers of Health, now the Society of Community Medicine, and "Towards an Occupational Health Service" in his Apothecaries lecture. His many honours included the Smith Award of the Royal Institute of Public Health and Hygiene in 1973 for "most noteworthy work in the discharge of his official duties". But no honour gave him greater pleasure than the Chairmanship of the Bristol Division of the British Medical Association and the Honorary Presidency of the Galenicals — the Medical Students' Society.

Robert Wofinden's professional life and work were devoted to the health and care of the people, whose every problem he illumined with a brilliantly penetrating mind and tackled with characteristic vision and formidable strength of purpose. How one could hear his delighted chuckle as one read the tribute in his obituary notice in The Times to "a ready pen and a gift of the gab", for he was a natural communicator and an inspiring teacher with a rare common touch; meticulously marshalling his facts but ever leading with the telling anecdote. To those who knew him only at second hand through the columns of the local Press or on the ubiquitous box he was the very embodiment of competence and uncommon sense: the personification of reassurance, trustworthiness and reconciliation. The M.O.H. was at his post: what ill could befall his domain?

In those last difficult years Robert Wofinden was physically stricken by a distressing and progressive disability. His indestructible courage and unfailing good humour transformed his illness into a quiet triumph of moral character and spiritual resourcefulness. With every little access of strength he would get out to visit a favourite spot or to call upon friends.

But Robert Wofinden, like all of us, would prefer to be remembered in full health and vigour. The citizens of this his adopted city and a whole generation of students will revere his memory. The immortality of his influence must be his lasting memorial.

## Figures and Tables

**Figure f1:**